# Mouse tracking performance: A new approach to analyzing continuous mouse tracking data

**DOI:** 10.3758/s13428-023-02210-5

**Published:** 2023-09-19

**Authors:** Tim Meyer, Arnold D. Kim, Michael Spivey, Jeff Yoshimi

**Affiliations:** 1 Department of Cognitive and Information Sciences, University of California, Merced, Merced, CA USA; 2 Department of Applied Mathematics, University of California, Merced, Merced, CA USA

**Keywords:** Mouse tracking, Movement dynamics, Singular value decomposition, Detrended fluctation analysis, Embodied cogntion, Complex systems

## Abstract

Mouse tracking is an important source of data in cognitive science. Most contemporary mouse tracking studies use binary-choice tasks and analyze the curvature or velocity of an individual mouse movement during an experimental trial as participants select from one of the two options. However, there are many types of mouse tracking data available beyond what is produced in a binary-choice task, including naturalistic data from web users. In order to utilize these data, cognitive scientists need tools that are robust to the lack of trial-by-trial structure in most normal computer tasks. We use singular value decomposition (SVD) and detrended fluctuation analysis (DFA) to analyze whole time series of unstructured mouse movement data. We also introduce a new technique for describing two-dimensional mouse traces as complex-valued time series, which allows SVD and DFA to be applied in a straightforward way without losing important spatial information. We find that there is useful information at the level of whole time series, and we use this information to predict performance in an online task. We also discuss how the implications of these results can advance the use of mouse tracking research in cognitive science.

## Introduction

Numerous experiments in multiple fields have shown that the body and environment have a profound influence on mental activity (McBeath, Shaffer, & Kaiser, 1995; Thelen, Schöner, Scheier, & Smith, 2001; Hotton & Yoshimi, 2011; Silberstein & Chemero, 2012). Ongoing, continuous exchange of information between body, mind, and environment suggests that measurements of any of these subsystems will produce information about cognition. This in turn predicts that cursor data, an indirect measurement of the body’s movements, will contain information about cognition. Mouse tracking studies can capture this data, and have many additional advantages. The “environment” in a computer task is highly controlled, and environmental variables can be monitored with high precision. The mouse, as a sensor for the body, can be measured with low latency. Computer tasks using mouse tracking data are relatively cheap and easy to produce, and data can be gathered from experiments run remotely over the internet.

**Fig. 1:**
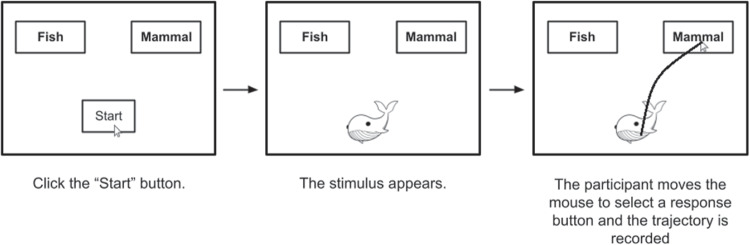
Standard mouse tracking task, based on the example in Hehman, Stolier, and Freeman (2015)

Most mouse tracking research follows a standard paradigm, a two-choice task in which a participant’s cursor begins at a predetermined start location, usually at the bottom-middle of the screen (Hehman, Stolier, & Freeman, 2015; Maldonado, Dunbar, & Chemla, 2019; Freeman, 2018). For a broad recent overview of this type of research see (Schoemann, O’Hora, Dale, & Scherbaum, 2020). The participant is then presented with a stimulus and asked to make a choice. They might be shown a picture of an animal and asked to identify that animal as a fish or a mammal by moving the mouse cursor to a text box labeled “fish” or “mammal” (See Fig. 1). As the participant moves their cursor towards the target, the (*x*, *y*) position of the cursor is recorded. The resulting vector of time stamped positions is a *cursor trajectory*. These cursor trajectories are then aggregated and analyzed (Hehman, Stolier, & Freeman, 2015; Stillman, Shen, & Ferguson, 2018).

In one of the most prominent examples of this type of research, participants are presented with either typical mammals such as cats, or atypical mammals such as whales. Participants’ cursor trajectories are found to be more curved during trials with atypical exemplars, suggesting a “graded competitive process” of categorization, rather than a serial model (Dale, Kehoe, & Spivey, 2007). Other prominent examples include: evidence for multiple distinct components of inhibitory control in Stroop and flanker tasks (Erb, Moher, Sobel, & Song, 2016), evidence against a dual system of emotion and reasoning in moral reasoning (Koop, 2013), evidence that better self-control facilitates quicker resolution of self-control conflicts shown by earlier changes in curvature of mouse movement (Gillebaart, Schneider, & De Ridder, 2016), evidence for “partial and parallel activation of stereotypes”, implying that “perceptual cues of the face” invoke multiple “simultaneously active stereotypes... and this mixture settles over time onto ultimate judgments” (Freeman & Ambady, 2009), and “evidence that cursor motion analysis has the capacity to predict emotional experience of the computer users" (Yamauchi & Xiao, 2018).

Despite its value in the study of continuous output during binary decision tasks, this research does have limitations. First, it is limited to serial tasks with a predetermined start and end point for each mouse movement. In addition, because the data are segmented into many discrete trials, dynamic processes which might build over longer timescales are hard to analyze. Thirdly, as is normal in many psychological paradigms, participants are required to follow rigid procedures in order to obtain clean data. These constraints make it difficult to apply standard mouse tracking techniques to *unconstrained mouse tracking data*, which is continuous over time, not segmented into individual movements, and not necessarily constrained in terms of starting position, end position, or preferred trajectory.

One way of dealing with the lack of structure in unconstrained mouse tracking data is to use machine learning techniques. Researchers in affective computing often use machine learning techniques to predict user characteristics (Kolakowska, 2013). However, though this approach addresses a particular business need, it does not usually provide results that generalize to other tasks, or provide novel scientific insights.

Another option is to draw on dynamical systems and complex systems approaches to cognition. For example, some have analyzed hand movements in open ended computer tasks such as corralling artificial agents in a computer game (Nalepka, Kallen, Chemero, Saltzman, & Richardson, 2017; Dotov, Nie, & Chemero, 2010). In tasks like these, where the movements are fluid and in continuous streams of motion, it is not clear what constitutes a single movement. Because of this, researchers in these areas tend not to decompose data into individual movements or behaviors, focusing instead on whole time series of behavioral data. One type of behavior that has been found when studying cognitive systems from this standpoint is power law relationships across multiple time scales. For example, several studies have found evidence for a 1/*f* power law in spectra characterizing human behavior when we look at an entire time series of a behavior, where *f* is frequency. A time series with a 1/*f* power spectral density is one in which the power spectrum is inversely proportional to the frequency of the signal (for example, lots of power at low frequencies, but low power at high frequencies). The relationship is a power law and thus it forms a straight line when plotted on log-log coordinates. Time series that are characterized by 1/*f* power spectra have long-range correlations that are thought to indicate an underlying interaction-dominant system. It has been found that much of the variance in psychology experiments exhibits a 1/*f* power spectrum, which suggests that humans are interaction-dominant systems where cognitive computations emerge from interactions among components rather than from inside any of those components (Van Orden, Holden, & Turvey, 2003). 1/*f* noise has since been found in many different domains from motor systems (Gilden, Thornton, & Mallon, 1995; Hausdorff et al., 1996) to music (Voss, 1975) to speech (Kello, Anderson, Holden, & Van Orden, 2008a). In addition, 1/*f* slopes can converge within distinct subsystems (indicating that the systems are coupled), i.e. key press and timing deviation in a rhythmic tapping task converge, and heart beat and pupil dilation converge as well, but central and autonomic nervous systems do not cross-converge within participants (Rigoli, Holman, Spivey, & Kello, 2014).

In this paper, we use two methods to study unconstrained mouse tracking data: singular value decomposition (SVD), and detrended fluctuation analysis (DFA). This pair of methods enables us to uncover structural and interpretable characteristics that predict performance in a task. SVD is an algorithm associated with principle components analysis (PCA), a standard method for high-dimensional data analysis and visualization used across a broad variety of domains (Eldén, 2007). Both SVD and PCA yield an ordered list of values (principal components, or singular values) that are associated with lower-dimensional subspaces that the data can be projected onto, in a way that reveals what the dominant characteristics of the data are. The values are ordered by how much of the variance in the data they explain, so that as one selects more of these components, they explain more of the data. The method can be applied directly to high-dimensional, complex-valued mouse trace data; is model free (it has no free parameters); reveals the most important components in a dataset in rank order; and can be used to develop interpretable diagnostics. SVD has not, to our knowledge, been applied to mouse tracking data, despite its advantages, but it has been used widely in cognitive science. For example, PCA has been used on motor movement data to show that fewer principal components are needed to explain the data when participants are engaged in a synchronized task (Riley, Richardson, Shockley, & Ramenzoni, 2011). PCA has also been used to discover that 75 percent of the variance between modalities in academic presentations (speech rate, intensity, slides changes, and gestural movement, etc) is accounted for by only 3 components (Alviar, Dale, & Galati, 2019), which correspond to different ways that presenters tend to speak. For example, the first component involved a positive relationship between speech rate, body movement, articulation rate and intensity, implying that ”speakers who tend to speak faster also tend to speak louder and move more.”

SVD is highly predictive and powerful, but it can be difficult to interpret. Thus, we also applied DFA, which has been independently applied to multi-scale data, and thus provides a baseline for comparison. With DFA a time series is broken into windows of decreasing sizes or scales, and within the windows at each scale, lines are fit to the data. The error in these lines is generally larger for the larger window sizes. The average error at each window size is plotted against window size in log-log, and a line is fitted to this data. The slope of this line is the Hurst parameter, which is a measure of fractal dimensionality in the data, which is associated multi-scale structure. It can be used to describe power law relationships and the color of noise, and is closely related to sample entropy. Researchers have used DFA to identify power law relationships between fluctuations in acceleration profiles for mouse movements at different time scales, and then shown that power laws change to reflect how well a person is “smoothly coping” with their mouse (Dotov et al., 2010). DFA has also been used on human inter-tap intervals when participants are attempting to tap along to a chaotic metronome to show that even though the metronome is chaotic and thus unpredictable, human inter-tap intervals will approximate the same statistical structure as the metronome (Stephen, Stepp, Dixon, & Turvey, 2008). This suggests that synchronization occurs not due to an internal tapping model with some error, but due to a more global process of coordination whereby the participant is becoming entrained with chaotic metronome.^1^

Several other researchers have considered techniques that might be applicable to the study of continuous mouse tracking data. For example, (Calcagnì, Lombardi, D’Alessandro, & Freuli, 2019) use a state-space model to describe mouse tracking data. The method is, however, validated against standard segmented mouse traces rather than unconstrained data, for example providing better fits to standard lexical decision data than other approaches. Others have used mixture models that treat mouse movements as combinations of simpler trajectories (Yu et al., 2007). These models have been used to identify neural correlates of components of motions. Neither technique has been applied to unconstrained mouse tracking data. We suspect SVD has some advantages over these approaches, in particular in being relatively parsimonious and straightforward to apply, but it remains to be seen in future research.

Summarizing: existing methods for analyzing mouse traces are focused almost entirely on segmented data (single mouse movements), while behavioral analysis techniques that can be applied to continuous movement have not, for the most part, been applied to unconstrained mouse data. A summary of these approaches to mouse tracking data, their standard use-cases, and their limitations relative to unconstrained mouse tracking data, is given in Table 1.

**Table 1:** Summary of existing methods for studying mouse traces, their typical uses, and their limitations relative to unconstrained mouse tracking data

Approach	Uses	Limitations
Curvature-based measures such as average deviation, AUC, and x-flips (Kieslich, Henninger, Wulff, Haslbeck, & Schulte-Mecklenbeck, 2019)	Measures straightness of movements, which is sometimes associated with decision conflict	Only applies to single movements
Sample entropy (Dale et al., 2007)	Predictability of the next sample after a set of samples	Does not capture multi-scale patterns
Measures of reaction time (Sternberg, 1969; Kieslich et al., 2019)	Associated with a variety of phenomena: attention, health, task demands, etc	Only applies to single movements
Derivative-based measures such as max velocity, average velocity, average acceleration, average jerk, etc. (Kieslich et al., 2019)	For examining fine-grained temporal dynamics of movements	Requires precise measurements, because taking the derivative of a time series amplifies its noise
Machine learning methods (Kolakowska, 2013)	Classifying and clustering types of trajectories	Hard to interpret or generalize from
Complex dynamical systems methods (1/*f* noise, DFA, max entropy, etc)	Measures global and multi-scale structure	Not applied to mouse tracking data thus far
Mixture methods (Yu et al., 2007)	Applies in principle to continuous movement and allows decomposition into constituent trajectories	Not applied to unconstrained mouse tracking data thus far
State space modeling (Calcagnì et al., 2019)	Provides good fits to data. Applies in principle to continuous movements	Not applied to unconstrained mouse tracking data thus far

In this paper we study unconstrained mouse tracking data in a simple clicking game similar to Whac-A-Mole. We used SVD and DFA to predict performance based on the mouse tracking data alone. Our results indicate that meaningful information exists at the level of an entire stream of mouse tracking data. In addition, we developed several novel approaches to analyzing mouse tracking data. First, we fit the mouse tracking coordinates to the complex plane. This allowed us to use information from both the *x* and *y* dimension simultaneously, rather than being constrained to one dimension, as is often done in mouse tracking studies. Second, we use SVD to define a diagnostic, $\eta$, which says how well players fit to an *accuracy space* defined by the principal components of the most accurate players. The quantity $\eta$ is continuously varying, explains more variance than DFA does, and can be used to predict a player’s accuracy in a way that is more interpretable than DFA.

## Methods

### Task

We designed a Whac-A-Mole game where several empty mole hills initially appear on the screen. Cartoon moles then appear and disappear in the mole hills in a pre-determined sequence (since this was an exploratory study, we wanted to minimize potential sources of variation). The player’s objective is to click the mole before it disappears and reappears in a different mole hill. A mole appears in a molehill for 650 ms before it disappears. If the mole is clicked, it changes to a cartoon picture of an unconscious mole for 350 ms. In both cases a mole then re-appears in another mole-hill. We pre-generated the random sequence of mole appearances so that every participant would experience the same pseudo-random sequence. The game ends after the participant has seen 120 moles (2-3 min). Upon completion of the game participants also filled out a short demographics form, and then were thanked and debriefed.

The game was built in javascript and played through the browser at a website.^2^ During the game we continuously collected participants’ cursor data, every $8-12$ ms (the maximum polling rate for javascript). We also collected participant’s click locations and recorded their accuracy in the game task.

### Participants

The experiment was deployed on Amazon Mechanical Turk (MTurk). We required that the MTurk workers have over a 95 percent approval rating, have not participated in one of our studies before, and be in the United States. We collected data from 600 participants in two samples. The first sample was collected on January 14, 2020 and had 300 participants. The second sample was collected on February 19, 2020 and had 300 participants. All participants were paid 35 cents for completing the 2-3 min study.

Three additional criteria were used to filter the data. First, we focused on mouse tracking data only, and thus removed those who did not report using a mouse (a question asked them what type of device they were using: mouse, trackpad, or other). This removed 83 people from the first sample and 102 people from the second sample. We did not require that they use a mouse explicitly to prevent participants from simply lying and saying they were using one when they were not. Second, we removed people who did not register sufficient cursor movement. Since javascript only polls mouse locations when the cursor is moving, if a participant simply let the mouse idle while the game ran or only tried to move the mouse a few times, the participant would not log many datapoints. We removed 2 participants from sample 1 and no participants from sample 2 for having less than 300 cursor locations reported. This also filtered participants who reported that they were using a mouse but were in fact using a touch screen or some other input device. Third, we included two catch questions in our demographics form: “how many letters are in the english alphabet” and “if you are reading this select the answer 17”. This eliminated 12 participants from sample 1 and 19 participants from sample 2. In all we removed 105 participants from sample 1 and 131 participants from sample 2 before analysis.

After filtering, demographics were as follows. For sample 1: 111 male, 84 female, mean age 40.11 ($\sigma = 21.35$), and approximately uniformly distributed experience with video games.^3^ For sample 2: 86 male, 83 female, mean age 40.22 ($\sigma = 13.17$), and also approximately uniformly distributed experience with video games.^4^

### Data collection and preprocessing

**Fig. 2:**
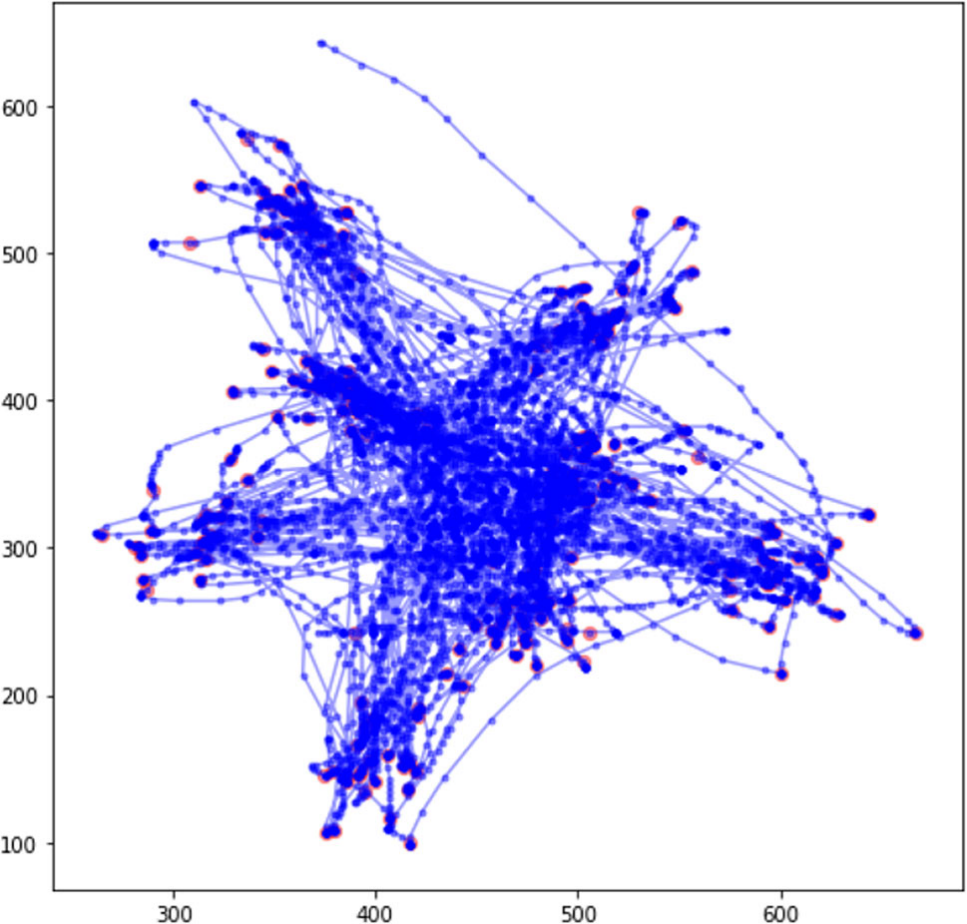
A sample mouse trace for one participant. Red dots correspond to mouse clicks

For each participant, the cursor position was collected throughout the task every $8-12$ ms, producing roughly 6000-component vectors of *x* and *y* coordinates, which is the mouse trace for that participant. An example of a mouse trace is shown in Fig. 2.

Most methods for analyzing time series assume one-dimensional data. However, mouse tracking data is inherently two-dimensional since it is samples the $x-$ and $y-$coordinates of the mouse position at discrete times. To accommodate this, researchers typically use only one dimension of their mouse trace data e.g., either the $x-$ or $y-$coordinate over time. However, this restriction potentially leads to information loss, especially if one does not have insight into which dimension is likely to carry the most information. As an alternative, we introduce a complex-valued time series $z_{n}$ where

$$\begin{aligned} z_{n} = x_{n} + \textrm{i} y_{n}, \quad n = 0, 1, 2, \cdots , N, \end{aligned}$$

with $x_{n}$ denoting the *x*-coordinate, $y_{n}$ denoting *y*-coordinate both at time level *n*, and $\textrm{i} = \sqrt{-1}$ denoting the imaginary constant. To isolate the $x-$coordinate, we evaluate the real part of $z_{n}$ and to isolate the $y-$coordinate, we evaluate the imaginary part of $z_{n}$. Thus, we can retain the full information available in the mouse trace by representing the two-dimensional mouse tracking data as a function of a single complex variable.

Mouse tracking through a browser has some inherent temporal variability as different computers and different browsers can poll at different speeds. To accommodate this we linearly interpolated all data using the pandas.resample method to make sure that all data points were exactly 20 ms apart and then trimmed all time series to the length of the shortest time series in the data set across both samples. (Several other resampling methods were tried to confirm that they did not impact the main results; since none did, we used the default method).

Since the mouse tracking data are resampled to be uniform in sampling rate and length across participants, we are able to compute, analyze, and compare their Fourier spectra. To perform this spectral analysis, we computed the discrete Fourier transform of the complex time series using numpy.fft.fft, to produce a spectrum $Z_{n}$ for each participant satisfying the relation

$$\begin{aligned} z(t_{j}) \approx \sum _{n = -N/2}^{N/2-1} Z_{n} e^{\textrm{i} 2 \pi f_{n} t_{j}}, \quad j = 1, \cdots , N, \end{aligned}$$

with $f_{n} = n/(N \Delta t)$ denoting the discrete frequencies and $\Delta t$ denoting the constant sampling rate.

One possible next step would be to compute derivatives of the time series in order to analyze velocity, acceleration, jerk, etc., which is common in mouse tracking research (Kieslich & Henninger, 2017).^5^ However, taking derivatives of mouse tracking data is inherently problematic for several reasons. First, computing derivatives of time series amplifies noise. Additionally, mouse tracking data is driven by an inherently discontinuous sampling process, which is exacerbated in online environments due to the browser (mouse positions are polled faster by the computer than they are by the browser) and network latency. These discontinuities lead to infinite derivatives. These issues can be dealt with if the number of samples is sufficient and appropriate filters are used (Nazir et al., 2008). Regardless, we show in our results below that we are able to recover valuable insight about information contained in mouse tracking data without needing to compute derivatives.

## Analysis and results

We start with a high-level sketch of how we applied SVD and DFA to our data. First, we pre-processed the data in the following steps:

- Import the data, which has already been filtered and converted into data frames (this is the publicly available data).
- Linearly interpolate the (*x*, *y*) coordinates for each individual person to ensure same-length time series and points equidistant in time.
- Create complex-valued coordinates from the (*x*, *y*) coordinates.
- Perform a Fourier transform on the complex-valued time series for each person.

We now have Fourier transforms for the complex-valued, interpolated time series for each participant. We then perform our SVD analysis, using the following steps:

- Split the data into high and low performance groups for Sample 1 and Sample 2.
- Compute the SVD of the Fourier transforms of the high performers in Sample 1.
- Create a space from a selection of the sample singular vectors from the high performers in Sample 1, using the most important vectors, accounting for about 50% of the variability in the data.
- Compare the high and low performing groups from Sample 2 to the space created from the singular vectors of the high performing group from Sample 1.

Note that we can use the space derived from Sample 1 to separate high and low performers in Sample 2, which implies a difference in behaviors that can be generalized across samples. We then investigated multi-scale structure in the data, using the following steps:

- Convert the spectrum to a power spectral density (PSD), and plot in log-log coordinates.
- Observe difference in the slopes of the PSD of the high and low performers.
- Apply DFA directly to participant mouse-traces.
- Use Hurst exponents of DFA to predict performance.

### Singular value decomposition to analyze performance

Let *P* denote the number of participants and $\textbf{Z}_{p}$ denote the vector of length *N* containing the values of the discrete Fourier spectrum for participant *p*. We form the $N \times P$ matrix *A* of complex numbers defined according to

$$\begin{aligned} A = \begin{bmatrix} \textbf{Z}_{1}&\cdots&\textbf{Z}_{P} \end{bmatrix}. \end{aligned}$$

In other words, the *p*th column of *A* corresponds to the discrete Fourier spectrum of the mouse tracking data for participant *p*. In what follows, we assume that $P < N$ since the number of participants in each study is smaller than the number of discrete Fourier frequencies.

The singular value decomposition (SVD) of the matrix *A* (Demmel, 1997; Trefethen & Bau III, 1997; Ramsay & Silverman, 2005) is

$$\begin{aligned} A = U \Sigma V^{*}. \end{aligned}$$

Here, the superscript $*$ denotes the complex conjugate transpose of the matrix. The columns of the $N \times N$ matrix *U* form an orthonormal basis for $\mathbb {C}^{N}$, the space of all complex vectors of length *N*. The columns of the $P \times P$ matrix *V* form an orthonormal basis for $\mathbb {C}^{P}$, the space of all complex vectors of length *P*. The $N \times P$ matrix $\Sigma$ has non-negative entries along its diagonal called the singular values, which we denote by $\sigma _{p}$ for $p = 1, \cdots , P$. The non-diagonal entries of $\Sigma$ are zero identically.

In fact, computing the SVD of the matrix *A* is the same as performing principal component analysis (PCA). We focus on the linear algebra interpretation of the SVD to study the discrete Fourier spectra of mouse tracking data. In particular we use concepts such as projections onto subspaces. By doing so we develop model-free methods that make use of any underlying algebraic structures in these data.

All matrices possess a singular value decomposition and the singular values are unique. The singular values are ordered by size,

$$\begin{aligned} \sigma _{1} \ge \sigma _{2} \ge \sigma _{3} \ge \cdots \ge \sigma _{P}. \end{aligned}$$

Let $\textbf{u}_{n}$ denote the *n*th column of *U* and $\textbf{v}_{p}$ denote the *p*th column of *V*. We can rewrite the SVD of *A* as

$$\begin{aligned} A = \sigma _{1} \textbf{u}_{1} \textbf{v}_{1}^{*} + \sigma _{2} \textbf{u}_{2} \textbf{v}_{2}^{*} + \cdots + \sigma _{P} \textbf{u}_{P} \textbf{v}_{P}^{*}. \end{aligned}$$

By writing *A* as this sum, we see that the singular values give a relative rank of the importance of the corresponding columns of *U* and *V* in the data – the first term gives the largest contribution, the second term gives the next largest, and so on. Additionally, we can consider approximations by truncating the sum above after some specified amount of terms. This approximation corresponds to the projection onto the subspace spanned by the vectors included in it.

Suppose we only use the first *k* singular values. Let $\tilde{U}$ denote the $N \times k$ matrix formed by taking the first *k* columns of *U* and removing the rest. The columns of $\tilde{U}$ form an orthogonal basis for a subspace of *A*, which we denote by $\tilde{\mathbb {U}}$. Now consider an individual participant’s discrete Fourier spectrum, $\textbf{Z}_p$. The projection of $\textbf{Z}_p$ onto $\tilde{\mathbb {U}}$ is given by $\tilde{U} \tilde{U}^{*} \textbf{Z}_p$. The length of this resulting vector is $\Vert \tilde{U} \tilde{U}^{*} \textbf{Z}_p \Vert$ with $\Vert \cdot \Vert$ denoting the Euclidean norm. When we compute

$$\begin{aligned} \eta = \frac{\Vert \tilde{U} \tilde{U}^{*} \textbf{Z}_p \Vert }{\Vert \textbf{Z}_p \Vert }, \end{aligned}$$

the value $0 \le \eta \le 1$ gives the fractional amount of $\textbf{Z}_p$ lying in the subspace $\tilde{\mathbb {U}}$. When $\eta = 1$, $\textbf{Z}_p$ lies entirely in $\tilde{\mathbb {U}}$. When $\eta = 0$, none of $\textbf{Z}_p$ lies in $\tilde{\mathbb {U}}$. We explain below how we use $\eta$ to study performance.

To analyze performance we first operationalized performance as accuracy in the game – the higher the percentage of moles clicked, the higher the accuracy and the better the performance. To investigate performance operationalized as accuracy, we first partitioned our data into “accurate” and “inaccurate” groups, where “accurate” participants scored above 50.5 percent, and “inaccurate” participants scored below 12 percent. These numbers were chosen to keep the group sizes about the same across the two samples. For sample 1 this resulted in 44 in the high accuracy group and 32 in the low accuracy group. For sample 2 high accuracy had 33 and low accuracy had 36. To address the worry that demographic differences in these splits accounts for our results, we regressed the demographic variables both on performance and on our predictor of performance, $\eta$. There was no meaningful relationship between any of the demographic variables and either performance or $\eta$.

**Fig. 3:**
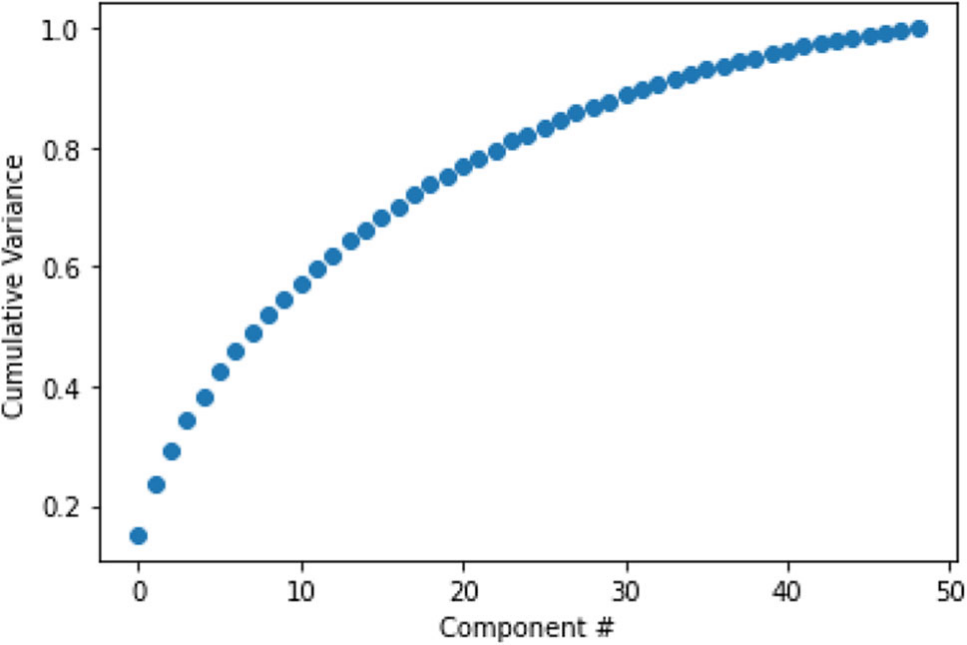
The cumulative variance accounted for by each component in the accuracy space. Notice that the first 8 components account for about $50 \%$ of the variance

We collect the discrete Fourier spectra of accurate players from Sample 1 and form the matrix *A* with them. Upon computing the SVD of *A*, we determine how many singular values are important in explaining the data. The singular values $\sigma _{p}$ for $p = 1, \cdots , P$ are proportional to the square root of the variance accounted for by the corresponding column of *U*. Thus, the cumulative sum of squares of the singular values is the cumulative sum of variance explained. This cumulative sum is shown in Fig. 3 and we determine from these results that $k = 9$ explains 50 percent of the variance. We call the resulting subspace $\tilde{\mathbb {U}}$ by considering the first 9 columns of *U* the *accuracy subspace*. By computing $\eta$, we determine the fractional amount a given discrete Fourier spectrum lies in the accuracy subspace. Consequently, $\eta$ is a measure of fitness to high performing players.

**Fig. 4:**
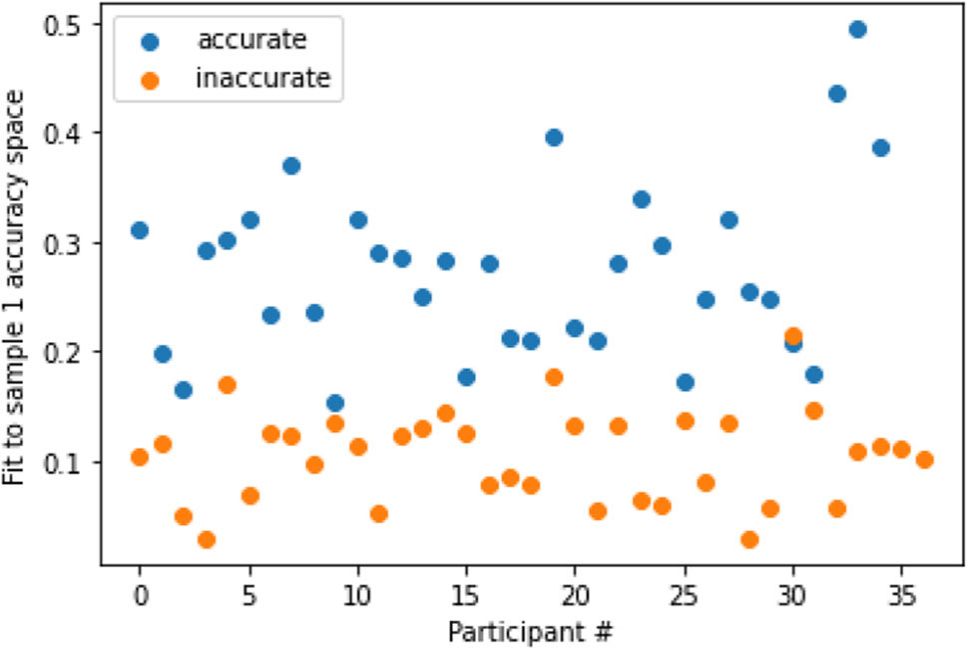
Projection of accurate and inaccurate participants in sample 2 to the accurate space from sample 1. Blue dots correspond to accurate players; orange dots to inaccurate players. The accurate players fit the space of accurate players from the earlier sample better with higher $\eta$ (which corresponds to how well a participant fits to a space). The two groups are also significantly different ($p<0.0001$). This shows that accurate and inaccurate players are separable using SVD

We consider results of Sample 2 to test how well the accuracy subspace from Sample 1 generalized to new, out-of sample participants. We identified accurate and inaccurate players in Sample 2 using the same criteria that we used to determine accurate and inaccurate players for Sample 1. To test out-of-sample performance, we computed $\eta$ for accurate and inaccurate players in Sample 2. The results of this computation are shown in Fig. 4. These results show that the two groups, accurate and inaccurate, are almost completely separable. They are shown to be significantly different according to a Welch’s t-test ($p<0.0001$). These results demonstrate the existence of structural features in the discrete Fourier spectra of accurate players that is not shared by less accurate players. The accuracy subspace contains algebraic structures inherent in accurate players that are not shared by less accurate players. Therefore, testing the extent to which a player’s discrete Fourier spectrum aligns with the accuracy subspace provides a diagnostic method for performance. Moreover, these results suggest that these structural differences persist across different samples.

**Fig. 5:**
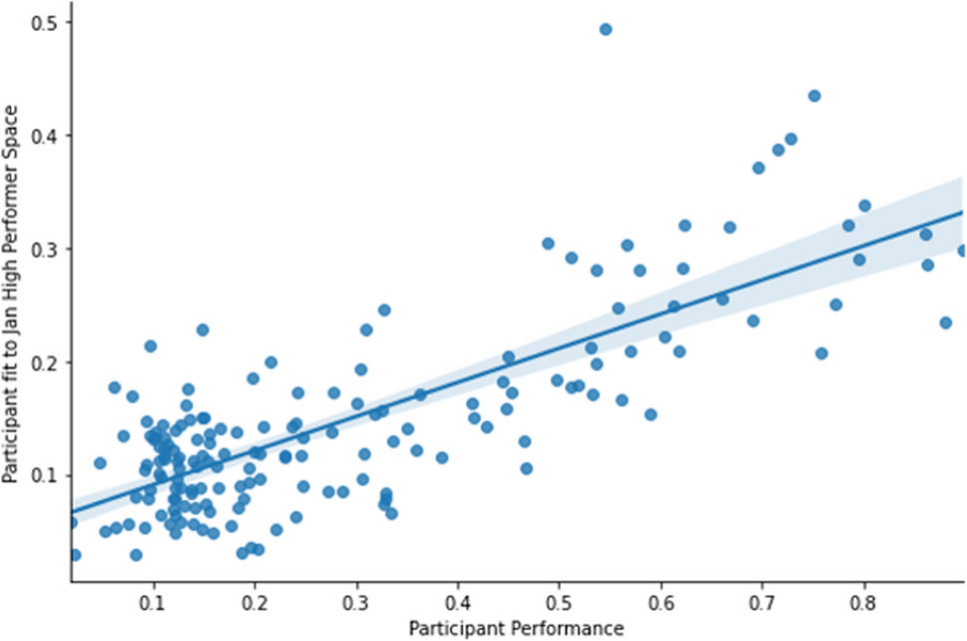
Performance regressed on fit to accuracy space. The relationship between accuracy and fit to the accurate space of sample 1 for participants from sample 2. Accuracy ranges from 0 (no mole hits) to 1 (every mole was hit). We can see that accuracy is related to fit to the accuracy space. The unstandardized $\beta$ is 0.30 with standard error 0.02. $R^2 = 0.61$ and $p<0.01$

Next, we computed $\eta$ for all players in Sample 2. In doing so, we found that there was a significant relationship ($p<0.01$) between accuracy and the value of $\eta$ (see Fig. 5) with $R^2 = 0.61$. Accuracy and fit both range from 0 to 1, and the unstandardized $\beta$ coefficient for fit regressed on accuracy was $\beta$ = 0.3 with standard error of .02, which means that as accuracy increases fit increases. These results show that the relationship does not just separate two groups of accurate and inaccurate players in the sample, but explains degrees of accuracy throughout the sample.

Representing our mouse traces using a single complex variable opens access and opportunity for novel methods of analysis. For example, we have been able to perform SVD/PCA directly on the discrete Fourier spectra of the full mouse tracking data. In doing so, we have been able to identify structural differences in the discrete Fourier spectra between accurate and less accurate players. We have found that these structural differences are statistically significant and persist in out-of-sample results.

### DFA analysis of performance

**Fig. 6:**
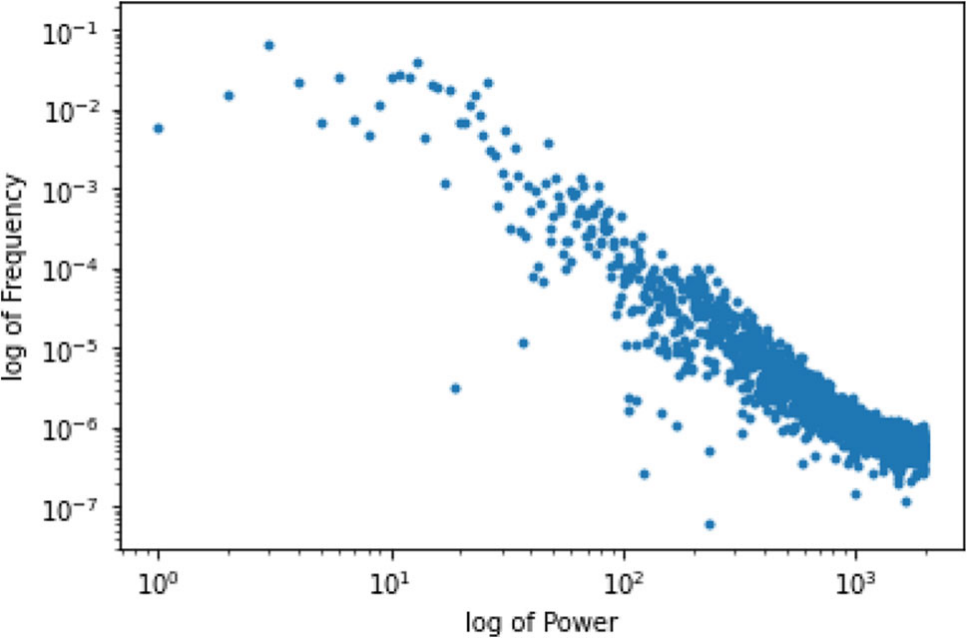
The most important component used for creating the accurate player space, plotted in log-log scale

To investigate the accuracy subspace $\tilde{\mathbb {U}}$ further, we consider the power spectrum of the columns of $\tilde{U}$ corresponding to the absolute value squared of each entry of a column of $\tilde{U}$. We then examine the shape of the power spectrum on a log-log scale. We have performed this analysis on the first 8 columns of $\tilde{U}$. Figure 6 shows the power spectrum for the first column of $\tilde{U}$ plotted in log-log scale. Over the first 8 columns of $\tilde{U}$, we observe a consistent linear structure to the power spectra. A linear trend for frequencies plotted in log-log can indicate a power law, which can in turn imply long-range correlations in a complex system. To investigate possible long-range correlations, we use DFA (Peng, Havlin, Stanley, & Goldberger, 1995).

DFA is a measure of the relationship between variance within windows of a time series and the size of those windows which, in turn, provides a measure of the amount of long-range correlation in time series (Stergiou & Decker, 2011). DFA has been applied in several areas of cognitive science (and extensively in other fields) as a tool to measure complexity in a time series. For example, Dotov et al use DFA to identify a change in the complexity of motor movements corresponding to a change from smoothly using an interface to cases of “breakdown”, where the user interface is perturbed so that it no longer behaves as the user expects it to Dotov et al., (2010).

To compute DFA we start with our complex time series:

$$\begin{aligned} z_1, z_2, \cdots , z_N. \end{aligned}$$

We center the data by subtracting the mean

$$\begin{aligned} \xi _n = z_n - \bar{z}, \quad n = 1, \cdots , N, \end{aligned}$$

and then compute the cumulative sum,

$$\begin{aligned} C_n = \sum _{j=1}^n \xi _j \quad n = 1, \cdots , N. \end{aligned}$$

We then partition the time series $C_{n}$ into windows of size $4 \le s \le N$. Using the smallest possible value, $s = 2$, is often not advised (Bryce & Sprague, 2012), so we set $s = 4$ as the minimum window size. For a fixed window of width *s* starting at *n*, we compute a least-squares regression model satisfying,

$$\begin{aligned} P_{d}(t_{n + j-1})\approx C_{n + j-1}, \quad j=1, \cdots , s, \end{aligned}$$

and then calculate the residuals,

$$\begin{aligned} r_{j} = C_{n + j-1} - P_{d}(t_{n + j-1}), \quad j=1, \cdots , s. \end{aligned}$$

Here $P_{d}(z)$ denotes the fitted polynomial of degree *d* (Shao, Gu, Jiang, Zhou, & Sornette, 2012). Linear fits are usually used so that in most applications (including ours), $d=1$.

We compute the root mean square of the residual for each window size *s*, to obtain the fluctuation value (Shao et al., 2012),

$$\begin{aligned} F_s = \left( \frac{1}{N} \sum _{t=1}^N | r_{n} |^2 \right) ^{1/2} \end{aligned}$$

Note that we are squaring the absolute values of the residuals, $| r_{n} |$ rather than the residuals themselves, since we are working with complex-valued time series.

We then fit a line to the relationship between the log-scaled $F_s$ and the log-scaled *s* (Shao et al., 2012). The slope of this line is $\alpha$, which is taken to approximate the Hurst parameter *H*. The Hurst parameter is a measure of fractal dimension in a time series. If $H < 0.5$ the process is considered to be anti-correlated in time such that high values tend to be followed by low values and vice versa. If $H = 0.5$ the process is not correlated in time, and if $1> H > 0.5$ then the process is said to be positively correlated in time (Ihlen, 2012; Nolds module — 0.5.2 Documentation, n.d.). However, if $\alpha > 1$ the process is non-stationary and can be modeled as fractional Brownian motion where the Hurst parameter of the systems is approximated by $H = \alpha - 1$ instead of $H = \alpha$ (Hardstone et al., 2012; Nolds module — 0.5.2 Documentation, n.d.). That is, for a non-stationary process, when $1> \alpha > 1.5$ the process is anti-correlated in time, if $\alpha = 1.5$ the process is not correlated in time, and if $2> \alpha > 1$ then the process is positively correlated in time.

**Fig. 7:**
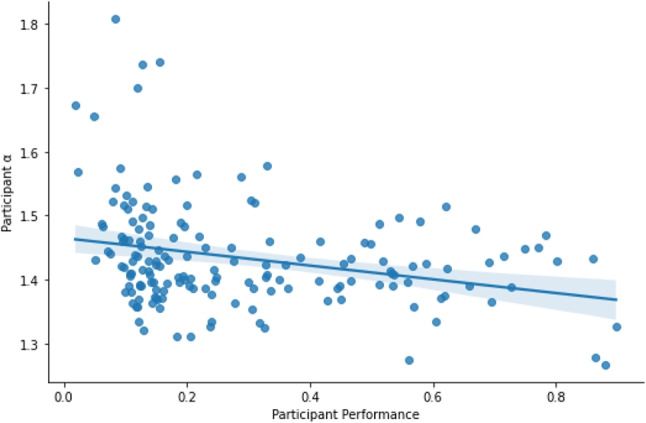
The parameter $\alpha$ regressed on performance. The negative slope $\beta$ = $-.75$ implies that the fractal dimension of the time series is decreasing as performance goes up. This in turn suggests that the time series are more likely to be anti-correlated as performance improves

DFA is a frequently used to analyze complex systems to determine the amount of long-range correlation in the data, which some contend is indicative of the degree of fractal structure in the system. The Hurst parameters provided by DFA were significantly predictive of accuracy ($p<.01$), as shown in Fig. 7. However, it was a much less powerful model with $R^2 =.08$ (vs. $R^2 = 0.61$ for SVD). Virtually all participants’ Hurst Parameters indicated some degree of nonstationairity in their fractal structure, as all participants were $\alpha > 1$. The negative relationship $\beta$ = $-.75$ implies that lower performing participants actually have more long range positive correlation in time than high performing participants, and high performers’ mouse movements are somewhat anti-correlated in time. This might seem strange at first, given that previous literature suggests that long-range correlations are positively related to performance. However, given that the moles’ next position is pseudorandom, it could also mean that participants whose Hurst parameters are below 1.5 are actually better approximating the target. In a similar fashion, expert Tetris players have been shown to rely heavily on the rotate button, allowing them to offload some of their cognition (Kirsh & Maglio, 1994). High-performing participants in this task appear to develop a pattern of interfacing with their environment that is substantially different from how the low-performing participants are interfacing with their environment. By exhibiting a lower fractal dimensionality in their time series, these high-performing participants are using up less of the “real estate” in the two-dimensional playing field and generating more efficient mouse traces that can be characterized in fewer fractal dimensions.

## Discussion

We designed a simple Whac-A-Mole like video game which participants played for a few minutes, during which mouse position data were captured. We did not know what might predict performance in this data, but were able to systematically explore it and find quantitative structures that were highly predictive of performance. Our results show that accurate players play differently than inaccurate players and that this difference is detectable in the mouse tracking data. Overall our efforts provide a good case study of how to explore and analyze unconstrained mouse tracking data. Even if a performance measure like accuracy were not available for a computer mouse task, these results show that those metrics could nicely be approximated by the mouse movements alone using principal components from known experts.

The SVD was substantially more predictive of accuracy than DFA ($R^2 = 0.61$ vs. $R^2 =.08$), suggesting it is more powerful as a means of predicting accuracy and other features of behavior. However, the DFA analysis is important because it has an interpretation in terms of cognitive science. DFA indicates the presence of multi-scale structure. In fact, the degree to which SVD outperforms DFA suggests that there is more going on for accuracy than just multi-scale structure.

Unconstrained mouse traces are difficult to analyze using standard technique such as reaction times (Table 1) because they lack well-defined beginnings and ends relative to individual mouse movements. This is representative of many real tasks outside of scripted psychology experiments. For example, in our data, mouse clicks can’t be interpreted as reactions to particular stimuli because we don’t know which stimulus participants are attending to. In some cases they may be attending to the currently visible mole when clicking, which is a straightforward reaction time. However, in other cases they might be predicting where a mole will appear next, adopting a strategy like “click in one place repeatedly to guarantee some hits”, or reacting to (and missing) a mole that is replaced by a new mole before clicking. In these cases the time interval between a mole’s initial appearance and the next mouse click is something other than a reaction time.

These methods and results provide two contributions to mouse tracking research and in particular to the analysis of unconstrained mouse traces. First, to our knowledge the only methods that have been used to analyze unconstrained mouse tracking data are machine learning techniques (Kolakowska, 2013; Liu, Fernando, & Rajapakse, 2018), which do not, however, provide interpretable results or generalizable insight into the mouse movements of the participants. As summarized in Table 1, other techniques could in principle be applied to this data, but this has not yet been done, to our knowledge. We have provided an initial guide to analyzing uninterrupted streams of mouse movements, in contexts where there are no clearly demarcated individual movements with determinate beginnings or endings. We have shown how to study unconstrained mouse tracking data in a way that is systematic, and can produce interpretable results. To be clear, the method we describe is not intended as a replacement for existing mouse tracking methods, which work well for single-movement data. Existing approaches to analyzing segmented mouse traces are clear, easy to interpret, and have an established track record.

Second, mouse tracking data naturally lies on a two-dimensional plane, but most time series analyses require a one-dimensional time series. The problem is usually solved by taking a time series of one dimension, such as the *x* dimension (Schulte-Mecklenbeck, Kuehberger, & Johnson, 2019). This solution works for binary forced choice tasks, where participants are making single movements and most of the information exists along one axis. However, even in these situations the one-dimensional analysis ends up neglecting potentially meaningful information in the other dimension, or in the combination of the dimensions. To address this we instead embed the data in the complex plane, which allows us to take two-dimensional coordinates and express them using a single complex variable. We are then able to convert our time series directly into the frequency domain. To our knowledge converting mouse coordinates to the complex plane has not been done before.

Outside of advancing methods in mouse tracking, we believe that our results help to characterize how high performers use their mouse. Our results indicate that behaviors associated with accurate game play produce long-range anti-correlations in the mouse movements. This result contrasts with some existing literature, which has found examples where long-range correlations are indicative of health or performance in human systems (Voytek et al., 2015; Hausdorff, 2007; Diniz et al., 2011). However, we could interpret accurate play in our task as attempting to synchronize spatially with a pseudo-random target, which does not have these long-range correlations. This is similar to the way participants attempting to synchronize with a chaotic metronome approximate its global multi-scale structure (Stephen et al., 2008). In the current experiment, rather than produce the long-range correlations they normally would in a repetitive task like walking or saying the same word repeatedly (Kello, Anderson, Holden, & Van Orden, 2008b), participants adapt themselves to the structure of the task. Additional hypothesis testing is needed to confirm this.

We believe this analysis could serve as a foundation for future research. In most natural settings user data cannot be precisely segmented into individual movements, and so traditional approaches to studying mouse movements cannot be applied. But there is often a desire to be able to gather data about such phenomena as affective states or engagement. These phenomena typically unfold at temporal scales much longer than individual mouse movements. For example, consider an immersive video game such as a first person shooter in a three-dimensional environment, which involves extended periods of play during which players provide continuous input to the game. Developers often want to know if changes they make to the game improve the experience of players, which is difficult to obtain via direct reporting. Methods like these could be used to study how players’ dynamics change in response to changes in the game, which could be indicative of player performance, engagement, team compatibility, ease of use, sentiment, etc. Any sufficiently large subset of mouse tracking data with some known characteristic could be used to define a space analogous to the accuracy space we used here. For example, data from players who report strong vs. weak team compatibility could be used to define a team compatibility space, which could then be used to identify new players for a team. An example where this type of approach is applied to measuring task engagement is Meyer (2022).

Future work extending this approach could also involve the development of an AI simulation that plays the Whac-A-Mole game. Different parameters and strategies could be programmed into the simulation and our approach could be tested for its ability to detect the presence or absence of those particular parameter settings and strategies.

This research could also provide insight into other components of cognition, such as response conflict, uncertainty, the time course of perception vs. cognition in tasks, etc., which have traditionally been a focus of mouse tracking research in cognitive science and psychology. To some extent, there is a paradigm difference between these approaches and ours. From our “embodied mind” and dynamical systems perspective (Dotov & Chemero, 2014; Kelso, 1995; Schmidt, Carello, & Turvey, 1990), we are revealing how spatiotemporal structures in behavior can be related to performance, based on complex profiles of long-term and short-term correlations. We are not looking for specific components of cognition in the data or “component-dominant dynamics” (Van Orden et al., 2003). Rather, we are looking at dynamic interactive patterns in the movement space, and making minimal assumptions about cognitive modules that may or may not be involved (Spivey, 2023). We are not ideologically opposed to componential studies, and could see future work integrating insights from both approaches. Our SVD approach allows an arbitrary data stream to be studied for its general characteristics, but this could then be used alongside more traditional studies, in the spirit of pluralism in cognitive science (Dale, Dietrich, & Chemero, 2009; Yoshimi, 2023).

Broadly speaking, the approach is more data driven than task or theory driven. The method is meant to identify complex patterns in a data set, and then to use these structures for analysis and prediction. The details will vary from task to task, and the results might not correspond directly to any existing theoretical constructs. But even if the approach is data driven, the methods are generalizable. The analysis pipeline we developed can be applied to any unconstrained behavioral task, and used to identify and interpret behaviors even when they are difficult to put into words. Consider soccer, basketball, or any team sport. Many features of these behaviors are the result of extremely complex interactions that have no pre-existing name or designation. Yet there is consensus among good players about best practices. As a result, a soccer teacher might just say “kick like *this*.” These methods can be used to identify such behaviors in time series data and to associate them with a space describing to what degree a person performs the behavior. Especially in online settings where such behaviors are common and the data relatively easy to gather, we expect these methods to be valuable.

## Conclusion

Using a simple online game we studied what information is available in unconstrained mouse traces. We used SVD to analyze the data, which allowed us to systematically explore it, while maintaining interpretability and building toward concrete hypotheses for future experiments. We found that the time series of mouse movements can reveal that accurate players play systematically differently than non-accurate players. In addition, the components of our SVD matrix factorization revealed that components which best-described accurate players had a power law structure. We then applied DFA and found that the behavior of accurate players is indeed characterized by a Hurst parameter that differs from that of inaccurate players. These findings also confirm the existence of high level information in mouse trace data. Thus there could be value in associating this type of data with more subjective and subtle states, such as levels of engagement, motivation, or affect.^6^

## Data Availability

The game code and python analysis scripts used in this study are available at https://github.com/potatchipsxp/MouseTracking. The data used by the python script is separately available at https://doi.org/10.5281/zenodo.5181395. Raw anonymized log files are available on request.

## Footnotes

1 DFA and other measures of complexity mentioned above are closely related. In the case of Diffusion Entropy analysis, for example, the output of both it and DFA bear the same inverse relationship to the scaling parameter of a Levy flight (Mariani et al., 2020).

2 The game is available here: *cogsci.us/TM/clickdamole*.

3 In response to the question “How much did you play video games growing up?”, with response bins of 0, $<1$, $1-2$, $2-4$, $4-8$, and $>8$, counts were 34, 13, 39, 29, 34, 46. In response to the question “How many hours of video games do you play each week?” with the same response bins, counts were 30, 42, 30, 31, 27, and 35.

4 In response to the question “How much did you play video games growing up?”, with response bins of 0, $<1$, $1-2$, $2-4$, $4-8$, and $>8$, counts were 26, 17, 23, 38, 23, 42. In response to the question “How many hours of video games do you play each week?” with the same response bins, counts were 28, 34, 28, 33, 21, 25.

5 With complex values, the *k*th derivative of a complex-valued time series has a convenient form: $z^{(k)}(t_{j}) \approx \sum _{n = -N/2}^{N/2-1} (\textrm{i} 2 \pi f_{n})^{k} Z_{n} e^{\textrm{i} 2 \pi f_{n} t_{j}}, \quad j = 1, \cdots , N$.

6 We thank Colin Holbrook, Ben Falandays, Chris Kello, Dave Noelle, Dan Holman, and Scott Hotton for their valuable feedback. We are grateful to Soraya Boza for Fig. 1.
